# Assessing the Role of Artificial Intelligence (AI) in Clinical Oncology: Utility of Machine Learning in Radiotherapy Target Volume Delineation

**DOI:** 10.3390/medicines5040131

**Published:** 2018-12-11

**Authors:** Ian S. Boon, Tracy P. T. Au Yong, Cheng S. Boon

**Affiliations:** 1 Department of Clinical Oncology, Leeds Cancer Centre, St James’s Institute of Oncology, Leeds Teaching Hospitals NHS Trust, Leeds LS9 7TF, UK; 2 Department of Radiology, Worcestershire Acute Hospitals NHS Trust, Worcester WR5 1DD, UK; tracy.ay@gmail.com; 3 Worcestershire Oncology Centre, Worcestershire Acute Hospitals NHS Trust, Worcester WR5 1DD, UK; cheng.boon@nhs.net

**Keywords:** artificial intelligence (AI), clinical oncology, deep learning, image guided radiotherapy (IGRT), intensity modulated radiotherapy (IMRT), machine learning, radiotherapy, stereotactic ablative radiotherapy (SABR), target volume delineation, volumetric modulated arc therapy (VMAT)

## Abstract

The fields of radiotherapy and clinical oncology have been rapidly changed by the advances of technology. Improvement in computer processing power and imaging quality heralded precision radiotherapy allowing radiotherapy to be delivered efficiently, safely and effectively for patient benefit. Artificial intelligence (AI) is an emerging field of computer science which uses computer models and algorithms to replicate human-like intelligence and perform specific tasks which offers a huge potential to healthcare. We reviewed and presented the history, evolution and advancement in the fields of radiotherapy, clinical oncology and machine learning. Radiotherapy target delineation is a complex task of outlining tumour and organ at risks volumes to allow accurate delivery of radiotherapy. We discussed the radiotherapy planning, treatment delivery and reviewed how technology can help with this challenging process. We explored the evidence and clinical application of machine learning to radiotherapy. We concluded on the challenges, possible future directions and potential collaborations to achieve better outcome for cancer patients.

## 1. Overview of Evolution and Advancement in Radiotherapy

Radiotherapy is a cornerstone curative treatment used either as a single modality or combined treatment in 40% of cancer patients. It is estimated that about half of all cancer patients will require radiotherapy treatment with a 2% increase in number of patients treated on a year on year basis in keeping with increasing incidence of cancer [1,2]. There is unequal access to radiotherapy geographically with regions in poverty having longer waiting times and less access to life saving radiotherapy [3].

The fields of clinical oncology and radiotherapy have witnessed and welcomed tremendous amounts of technological advancement since its infancy and the discovery of radioactivity by Marie Curie 150 years ago [4]. Radiotherapy, in its basic form, uses ionising radiation to deposit energy to destroy cancer cells. Radiotherapy is now delivered in various methods including external beam therapy using linear particle accelerators (Linac) which uses photons, electrons and protons. Internal forms of radiotherapy delivery include brachytherapy with application of internal or intracavitary treatments and delivery of radioactive substance, such as application of radioiodine in thyroid cancer.

Clinical practice of radiotherapy has always varied nationally and internationally [5]. At its infancy, radiotherapy did not start as an exact science but evolved through clinical acumen, innovation and evidence-based research [4,5]. The field of radiobiology is an attempt to model and explain the effects of radiotherapy treatments. Modern radiotherapy trials and research are rigorous, well designed and have led to much practice changing contribution to cancer patients and improving cancer outcomes [4].

Moore’s Law coined after George Moore who first observed that in every two years the amount of transistor which could be incorporated into an integrated circuit double [6]. The exponential growth of computer processing power with technological advancement has enabled the arrival of precision radiotherapy. Newer techniques allow radiotherapy to be delivered ever more accurately and effectively thereby achieving the goal of increasing the therapeutic ratio with better cure rates and lesser side effects from treatment.

Modern radiotherapy techniques with intensity modulated radiotherapy treatment (IMRT) allows clinicians to manipulate radiotherapy beam delivery to conform to various shapes of cancer thereby reducing side effects to adjacent normal organs [7]. Further advancement of this technique subsequently gave rise to volumetric modulated arc therapy (VMAT) which allows even better dose conformity and further reduction of treatment duration. VMAT achieves this by allowing concomitant variation of parameters such as dose rate, speed of radiotherapy machine gantry rotation and movement of multileaf collimator during treatment delivery [8].

Image-guided radiotherapy (IGRT) allows clinical oncologists to account for the fourth dimension of time in radiotherapy delivery allowing real-time tracking of tumours with ever more accurate treatment and further preservation of critical organ at risk that maybe in field of treatment. This then led to the birth of stereotactic body radiotherapy (SBRT) or stereotactic ablative radiotherapy (SABR) which allows the delivery of even higher doses of radiation than previously possible in fewer fractions of treatment to allow the complete ablation of discrete, small volume cancer primary or secondaries (oligometastases) [7]. This precise delivery of above the normal doses of radiotherapy has not resulted in significant increased side effect of normal organs in treatment areas. SABR has been successfully introduced to early lung cancer allowing treatment options in patient unsuitable for surgical options and as a result increasing number of clinical trials has begun exploring the role of SABR in re-irradiation, oligometastases (three or fewer sites of secondary cancer) and hepatocellular carcinoma [9,10]. The use of such technique for brain metastases has been termed Stereotactic radiosurgery (SRS) and has increasing clinical indications offering treatment options to cancer patients where there were none previously [11].

The next era of radiotherapy advancement is the advent of proton beam therapy. This shift towards using proton instead of photon as treatment particle confers the benefit of the inherent physical properties of proton dose distribution described as Bragg peak effect. As a photon traverses normal tissue to reach the cancer target, it loses energy along the way in accordance to inverse square law and with least dose deposition towards the end of transit. Proton ionising radiation delivers a heavier charged particle, has a peak dose deposition towards the end of transit which subsequently tails off very rapidly. This property is exploited and thought to be able to deliver radiotherapy more effectively and sparing more critical organ at risks (OAR). The logistics of this approach and evidence is still being generated with the start of nationally funded proton beam centres in the United Kingdom to conduct patient trials [12].

Better imaging with magnetic resonance (MR) has allowed superior soft tissue resolution potentially further improving radiotherapy delivery by inputting MR images into radiotherapy planning. The feasibility of magnetic resonance-guided radiotherapy is actively explored in the trials and research setting with most progress in prostate cancer [13].

With the constant introduction of novel technology available, clinicians and on a greater level, cancer healthcare services often have to decide on how to approach these technological advancements while justifying spending limited financial resources. One approach has been to link up regional cancer centres and set up large cancer networks to collaborate and share both the costs and possible benefits of adaptation of such modern radiotherapy advancement though the clinical trials and research effort [4,14].

## 2. Radiotherapy Planning, Target Volume Delineation and Quality Assurance

Central to the radiotherapy planning process is radiotherapy target delineation or contouring. Clinical oncologists obtain up-to-date relevant clinical images and uses computer software to manually outline cancer sites to deliver radiotherapy dose. Guided by clinical information, cancer pathology and diagnostic images, the visible cancer volume is outline as gross tumour volume (GTV). Clinicians then expand the volume by adding margins to take into account of potential spread of the cancer to delineate the clinical treatment volume (CTV). Further additional margins are added to form the final planning target volume (PTV). The PTV adds a margin of safety to account for potential uncertainties during treatment planning and delivery taking into account the adjacent normal tissues [15].

Equally essential is the contouring of organ at risks (OAR) or normal tissues which are areas to limit or avoid doses of radiotherapy to avoid side effect of treatment [15]. Clinical models, such as the normal tissue complication probability (NTCP) model have been developed to help clinicians in predicting the tolerable radiotherapy doses that can be delivered to normal tissues and account for differences in normal tissue radiosensitivity [16]. These roles and responsibilities within the United Kingdom are governed by the legal framework which clinical practitioners must abide [17].

The radiotherapy planning process is highly complex, and where clinician judgement is required, there is the potential of introducing error. Error in this process can be disastrous to cancer patients as it results in missing the tumour or delivering harmful doses of radiotherapy to normal tissue resulting in potentially life-threatening side effects. This is even more pressing in the era of precision radiotherapy, where margin of treatments are often tighter; therefore, only a small margin of error would result in geographical miss of tumours. A retrospective study in head and neck cancer to evaluate the recurrence pattern in using an IMRT technique has revealed treatment failures arise as a result of poor target delineation [18].

Variation in radiotherapy target delineation exists among clinicians from centre to centre nationally and internationally [19,20]. It will be difficult to eliminate variation completely, as there are no specific tests that will ascertain the CTV for a particular tumour volume is absolutely accurate. In tumour sites or individual patient anatomy variations where there is no protocol or consensus, a systematic approach to peer review process can help with standardisation and reduce variation in target volume delineation amongst clinicians [21]. Introduction of standardised atlas agreed by head and neck cancer multidisciplinary clinicians in contouring has been shown to reduce individual variations in target volume delineation [22].

Quality assurance (QA) and peer review are paramount to ensure delivery of quality radiotherapy treatments, detection and reporting of errors. This should be standard practice for oncologists to establish a safe culture for radiotherapy delivery [23]. Continuous education for trainees and fully registered practitioners is essential towards achieving this [21,24].

## 3. Artificial Intelligence, Machine Learning and Deep Learning

**Figure 1:**
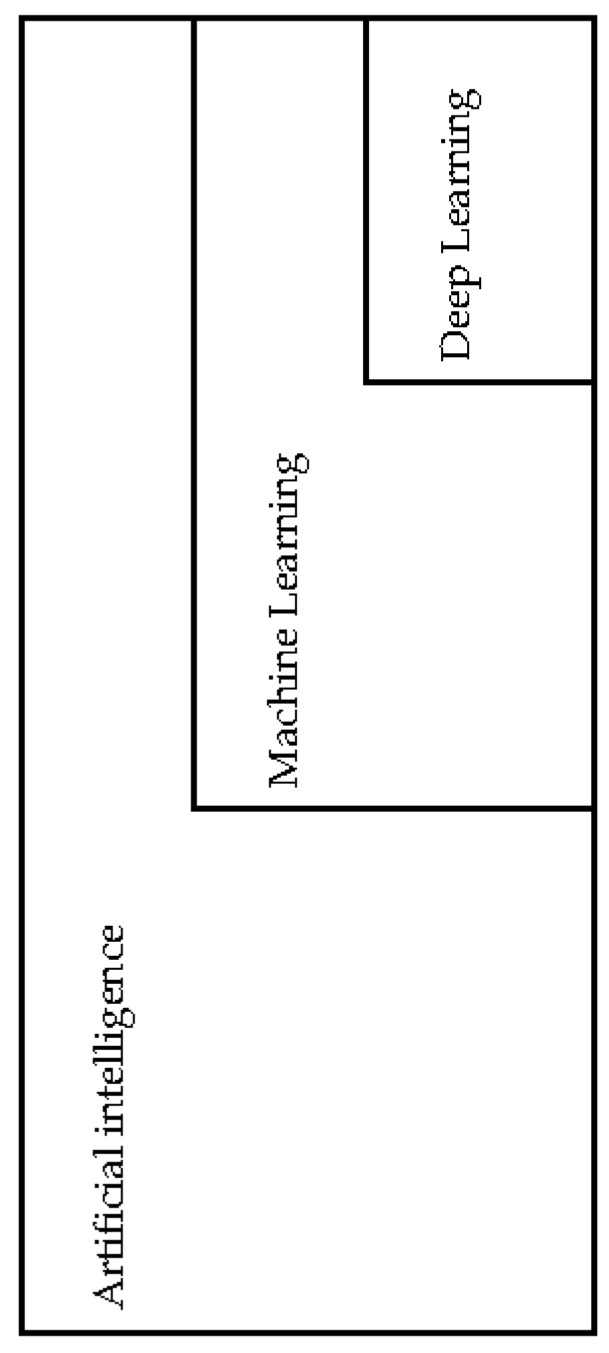
A diagrammatic representation in an attempt to reflect the overlapping domains and relationship of the fields of artificial intelligence, machine learning and deep learning.

Artificial intelligence (AI) is a branch of computer science that attempts to emulate human-like intelligence in machines through the use of computer software and algorithms without direct human stimuli to perform specific tasks [25,26]. This relatively young field began in the 1950s and has received waxing and waning attention through time. In the 1980s, there was a period termed the “AI winter” where there were significant reductions of financial interest and attention to this field. The revival of interest in AI began in 2000. The rise of computation power and reduction of financial barriers led to the birth of the latest field of deep learning. Figure 1 below gives an approximation of the overlapping domains and relationships of the fields of AI, machine learning and deep learning [25,26].

ML uses computer software, modelling and algorithms to detect patterns and correlation through the learning process by providing it with databases of raw data. Machine learning approach has been applied to oncology and radiotherapy. The relevant ML methods that have been applied to radiotherapy target delineation are Support Vector Machine (SVM), Artificial Neural Network (ANN) and the latest variant deep learning method (DL) [27].

In radiotherapy specific data modelling, Cox-regression is used for survival analysis and qualitative data such as treatment side effects are analysed using linear regression. The SVM model is used to analyse multiple linear and non-linear variables which it transforms into vectors and processes this to detect correlation or predict outcomes [27]. This method has been used to predict sensitivity of oesophageal cancer patients to chemo-radiotherapy by analysing blood markers [28] and to predict treatment outcome in lung cancer patients treated with SABR radiotherapy [29].

ANN is a machine learning approach that models after the human nervous system. Each “neuron” receives an input and creates an output in successive networks and it can assign the importance to a particular connection. ANN is made of multi-layered neural networks connected allowing the model to assign the degree of importance randomly to the processes until it detects a connection or correlation. ANN has been used in predicting outcomes of radical radiotherapy in prostate cancer [30] and head and neck cancer treated with chemo-radiotherapy to a good degree of accuracy [31].

Deep learning (DL) is the latest form of ML. It is a variant of ANN that is made of a much greater number of hidden layers or multi-layered processing networks [26]. DL is capable of both supervised and unsupervised learning. In essence, the DL approach uses multiple levels of abstraction in its process to learn connections and correlations when provided with a database of data. It is a repetitive process requiring time to train, large amounts of computation power and a database large enough for it to learning process [27]. The DL method has been applied to radiotherapy target delineation of OAR in head and neck cancer [32].

## 4. Application of Machine Learning in Radiotherapy Target Delineation

The process of manual contouring of tumour volumes and OARs in the radiotherapy target delineation process is a complex, labour intensive and time-consuming process. ML approach has been explored to help with this process by way of auto-delineate or producing auto-contours either to the tumour or normal structures.

Commercially available software to help with automatic segmentation has been available for a number of years and are predominantly divided into the atlas-based segmentation (ABS) or used in combination with model-based segmentation (MBS) [33]. Computed tomography (CT)-based radiological images of patient are deformed incorporating information of organs to be segmented which is then processed with algorithms to process a final combined contour which gives clinicians a best representation of a patient’s atlas to work on [34].

Recently, ML and DL methods have also been used to auto-delineate radiotherapy planning. A review paper on auto-segmentation use in head and neck cancer shows six out of ten studies reporting clinician time-saving in using such software, but one paper did not [35]. Assessment of the quality of contours was mixed but the review showed that the software were not capable of producing outputs that can be used clinically as yet.

Comparisons between contour outputs of auto-segmentation software can often be difficult. In order to compare a specific contour against another, a statistical tool has been postulated and named as the Dice similarity coefficient (DSC) or Sørensen–Dice coefficient [36]. It is a statistical method of comparing spatial image and the performance of auto-segmentation software by comparing the product contours and there has been many refinements of this tool.

A recent UK-based research group has used the deep learning method to explore contouring OAR in head and neck cancer. This study, performed on over 663 patient radiology and contour, has compared the performance of ML against the manual contours done by senior radiographers and results adjudicated by consultant clinical oncologist has shown reporting achievement of clinically acceptable contouring when compared to senior radiographer of OAR structures except for brainstem and right lens [32].

Deep learning-based contouring of tumours in head and neck cancer based on MR images has also shown good degree of agreement to clinician manual contours [37] and also used to delineate high risk CTV in CT based head and neck planning [38]. A study presented in the abstract form on the application of ML to head and neck contouring of OAR has now used it in clinical practice after retrospective and subsequently prospective validation. They reported that 50% of the auto-contours are used clinically without changes with their multi-atlas-based auto-segmentation further saving clinician time [39]. This approach has also been replicated to compare clinician edited auto-contours against manual contouring in head and neck cancer with saving up to 112 min per plan with clinically acceptable output contours [40].

Such approaches have also been explored in prostate cancer [41], lung cancer [42] and breast cancer [43]. Although the ML and DL approaches are making further gains in saving clinician time and producing good quality contours, we are not at the stage where such software can replace clinician function. However, there are clear benefits and roles for ML approaches in radiotherapy target delineation, particularly in performing specific repetitive tasks such as contouring simple replicable organs at risks and coming up with radiotherapy contours that can save clinician time and increase productivity. Table 1 below summarises the radiotherapy target volume definition, radiotherapy modality, validation method and major outcomes of eight relevant publications pertaining to machine learning application to radiotherapy target volume delineation.

## 5. Application of Machine Learning in Radiotherapy Delivery and Image Guided Radiotherapy

At present, many modern radiotherapy linear particle accelerators (Linac) have the capacity to perform daily “cone-beam” CT scans that use megavoltage X-rays for treatment verification [23]. These images have poor discrimination of soft tissue structures but, as these images are utilised to match the treatment plans to patients’ day to day anatomy and reduce intra-fractional shifts, it is considered acceptable for current usage for image guided radiotherapy.

If the on-set cone-beam megavoltage CT scans does show significant anatomical changes such as weight loss or tumour movement (particularly in head and neck radiotherapy plans), this will trigger a manual decision to repeat a conventional CT planning scan using kilovoltage X-rays. These kilovoltage X-ray CT scans will have sufficient contrast between soft tissue structures to allow re-contouring and re-planning of each patient’s radiotherapy plans.

Each of these steps will require significant manual input, in the case of daily reviews of the cone-beam images, by at least one (1), if not two (2) experienced treatment radiographer(s) to review each cone-beam image daily prior to delivery of the radiotherapy dose. If the said radiographer(s) were to notice significant anatomical mismatches, this will trigger either a senior radiographer, medical physicist or consultant clinical oncologist (or all of them) to decide on the suitability of treatment delivery or to delay and repeat kilovoltage X-ray CT planning scans. Each of these steps delays patient treatments and results in significant increase in workload for the department.

In addition, with the development of magnetic resonance imaging technology combined with conventional linear accelerators [13], the ability of technology to generate vast quantities of additional imaging data will require extra training for treatment radiographers, medical physicists and consultant clinical oncologists in order to safely utilise these newer technologies, let alone for the benefit to accrue to patients. This all opens the path to have machine learning growing in parallel with the training programme for medical and allied health professional staff.

The ability of current staff to cope with the growing workload as well as to innovate and benefit from modern technology is limited by access to sufficient human resources. Humans working together with machine learning will shorten the time needed to train staff as the algorithm can “learn” as well as train staff. This is best demonstrated by the rapid developments of modern chess players in rapidly learning hundreds of years of chess developments in a matter of months by repetitively training against the best computer AI programmes. A deep learning programme trained only for hours from first principle to play chess has managed to attain world champion status [44].

## 6. Opportunity and Challenges to Application of Machine Learning Approach to Oncology and Radiotherapy

The United Kingdom National Health Service (NHS) is actively exploring the evidence for AI and generating a strategy for adapting and application of AI to contribute and optimise patient care [45]. The potential application of AI has been subject of much interest to the radiology and oncology community [46,47]. The United Kingdom Royal College of Radiologists (RCR) has identified AI as one of the most significant technological advancement in healthcare. The RCR recognises AI as having the capacity to reshape the specialty and has proactively sought all stakeholders to address the challenges of applying this to patient care. Legal framework, governance, quality assurance and staff training are some of the vital issues that must be explored prior to applying such technology to clinical practice backed by robust evidence-based cost benefit analysis [46].

Other applications of ML to the field of oncology includes initiatives are to harness the predictive capability of machine learning with regards to oncology [48]. Progress has been made in using ML approach to predict cancer recurrence namely in breast cancer [49], oral squamous cell carcinoma [50] and cervical cancer [51]. The progress made in these cancer sites reflects the availability of large datasets available in these cancer sites for the ML approach to train their modelling. A breast cancer recurrence predictive model has recorded high specificity, sensitivity, negative predictive and positive predictive values [49]. It claims to even surpass the current model used in routine breast cancer practice, PREDICT [52], although this requires further validation. Attempts to use ML to predict cancer survival has also been made in breast cancer [53], lung cancer [54] and others still requiring further work and validation. ML can also be potentially used to predict the need of adaptive radiotherapy due to changes in anatomy though treatment. Early retrospective work has been explored in head and neck cancer to predict the need to re-plan radiotherapy plan due to changes of radiotherapy doses to parotid glands through treatment [55].

The ML approach has also been explored to help with radiotherapy quality assurance. A predictive model has been applied to help with the quality assurance of IMRT plans which has been able to detect deviations of up to 3% and set up errors with a good discrimination [56]. A similar approach has also been explored for quality assurance process of VMAT radiotherapy plans for prostate cancer. This model uses a dataset of previous dose-volume histogram (DVH) of the VMAT prostate plans to detect deviations or delivery errors [57].

Challenge remains in applying ML to oncology and radiotherapy workflow process. AI may be able to perform specific tasks such as detect specific organ structures on radiological images but how it interprets information is different to human cognition. AI would be capable of detecting structures that cannot be normally seen by the human eye, but such capacity maybe of no clinical utility and it can be fooled to detect incorrect organ structures without realising it [58]. The derivation of the exact algorithms used in AI is often not well described or devoid of clinical reasoning but guided by intuition and empirical learning [27]. Scarce information of the algorithms used is available in the public domain maybe due to being developed by private initiatives.

Machine learning is an iterative process of detecting links through computer models and algorithms and requires a huge database of raw data for its learning process. Cancer images, radiology data and patient radiotherapy contours are often stored in secure servers that are not linked in local hospital setups [47]. The huge barrier for advancement of machine learning in radiotherapy would be the logistics of accessing such radiotherapy databases and diagnostic images of sufficient quality for machine learning training. Consideration of patient confidentially and data security would also have to explored.

Comparison of the end points, performance and efficacy of machine learning-derived auto-contouring software capability can also prove to be challenging given the complex nature of this process. Recommendations has been made in order to standardise and validate such capability by assess three major domains such as ontology (capability to specify parameters including specific tumour sites, radiotherapy delineation protocol and patient-specific criteria), performance evaluation and benchmarking assessment [59].

## 7. Conclusions

Machine learning has and will probably continue to evolve rapidly with wide-ranging application to oncology and radiotherapy. Most of the software and models for radiotherapy target delineation or oncology available at this point in time are not yet used in the routine clinical setting, although improvements are expected with time. There are roles for AI in radiotherapy target delineation such as repetitive tasks, contouring of reproducible organ structures and others but it will be with clinician oversight.

Prior to any application to healthcare, AI needs to be validated, tested for reliability and have a robust quality assurance before it can gain the confidence of clinicians and patients to be introduced safely to routine clinical use. Understanding both the capability and, equally essential, the limitation and potential failures of AI is paramount before adapting AI into healthcare.

Crosstalk between computer scientists and oncologists is essential to ensure the full capability and potential of the technology is achieved. Both fields are very complex with the needs of oncologist often not known to computer scientists while the skills and capability of computer scientists can often be obscured by the nuances of computer science to the medical community. Close collaboration is essential so that we are not revisiting another “AI winter” but rather an “AI spring” heralding to a new dawn of digital healthcare for the benefit of our patients.

## Figures and Tables

**Table 1:** Relevant publications on machine learning approaches to radiotherapy target delineation. Abbreviations: organ at risk (OAR), computed tomography (CT), Dice similarity coefficient (DSC), magnetic resonance imaging (MRI), clinical target volume (CTV).

Publication	Cancer Site	Machine Learning Method	Target Volume Delineation	Radiotherapy Planning Modality	Number of Patients	Validation	Outcome and Important Features
Nikolov S [32]	Head and neck	Deep Learning	OAR	CT	663	Compared against manual contours by senior radiographers adjudicated by senior consultant clinical oncologist	19 out of 21 OAR surface DSC scores less than 5% deviation when compared to clinician manual contours. Did not achieved target for brainstem and right lens
Li Q [37]	Head and neck	Deep Learning	Tumour	MRI	29	Compared against manual contours by consultant clinical oncologists	Mean DSC 0.89. Good agreement when compared to manual contours
Cardenas CE [38]	Head and neck	Deep Learning	High risk CTV	CT	52	Compared against manual contours by clinicians	Median DSC 0.81. Good agreement when compared to manual contours by clinicians with only minor or no change
McCarroll R [39]	Head and neck	Machine Learning	OAR	CT	128	Compared against manual contours by consultant clinical oncologist	Mean DSC 0.78. Once validated was used in clinical setting and prospectively tested with accuracy of 63%. 50% of auto-contours were used without changes
Speight R [40]	Head and neck	Machine Learning	CTV	CT	15	Auto-contours edited by clinicians compared against manual contours by clinician	Edited CTV DSC 0.87. Mean clinician time saved by 112 min per plan when compared to manual contours
Martin S [41]	Prostate	Machine Learning	Tumour	MRI	15	Compared against manual contours by 5 clinicians of varying experience	3 phases of trial. Mean DSC 0.89. Good agreement with clinician contours requiring minimal changes. Time saved in all cases
Lustberg T [42]	Lung	Deep Learning	OAR	CT	20	Compared against manual contours by a single radiotherapy technician	Median DSC 0.57 and median time saved by 79%. Saved time in lung and spinal cord contouring but not for left lung and oesophagus
Bell LR [43]	Breast	Machine Learning	Tumour	CT	28	Compared against manual contours by 8 clinicians	DSC more than 0.70. Good agreement with clinician manual contours. Coverage agreement poorest towards heart border structures
